# Supplementary material for: Tumour-associated missense mutations in the dMi-2 ATPase alters nucleosome remodelling properties in a mutation-specific manner
Source: Nat Commun. 2018 May 29;9:2112. doi: 10.1038/s41467-018-04503-2 (PMC5974244; doi:10.1038/s41467-018-04503-2)
Supplement: Supplementary file 1 — Supplementary Information [file 41467_2018_4503_MOESM1_ESM.pdf]

Tumour-associated missense mutations in the dMi-2 ATPase alters nucleosome remodelling properties in a mutation-specific manner

Kovač *et al.*

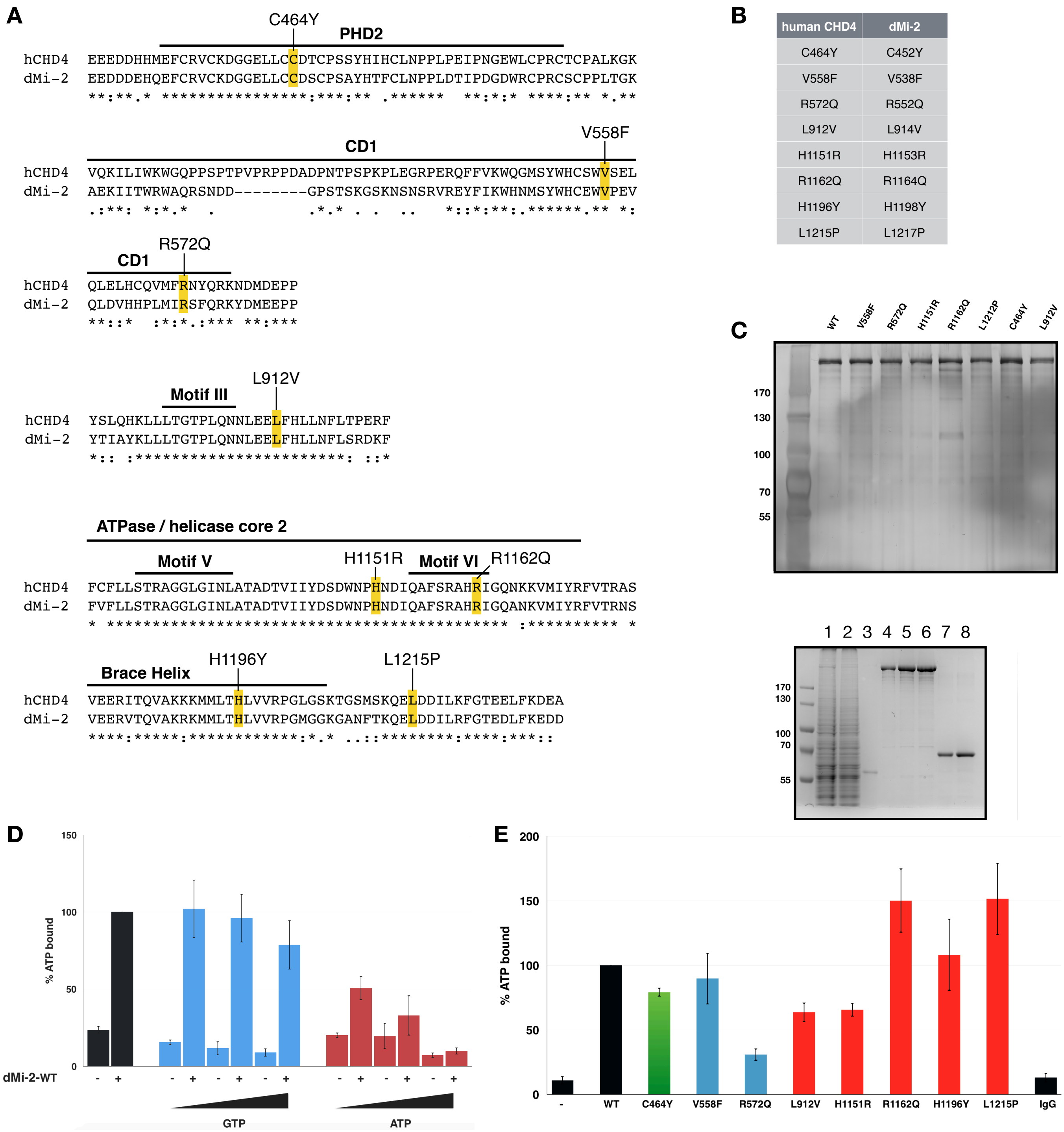

Supplementary Figure 1. (A) Alignment of *H.sapiens* CHD4 and *D.melanogaster* Mi-2 sequences surrounding the mutations analysed in this study. Mutated residues are highlighted in yellow. Domains and motifs are indicated on top. **(B)** Table of mutations analysed in this study. Left: the CHD4 mutations identified in endometrial cancer. Right: corresponding mutations in dMi-2. Note that throughout the manuscript the numbering for CHD4 is used even when discussing dMi-2 mutants. **(C)** Recombinant dMi-2 proteins. Top panel: 1μg of immunoaffinity-purified, FLAG-tagged wild type (WT) and mutant dMi-2 proteins resolved by SDS-PAGE and visualised by silver staining. Mutants are indicated on top. Molecular mass markers are shown on the left. Bottom panel: Purification of immunopurified FLAG-tagged dMi-2 H1196Y mutant. Lane 1: 1% of Sf9 extract used for purification (input); lane 2: 1% of flowthrough; lane 3: eluted anti-FLAG beads (1%), lane 4: 1μL FLAG peptide eluate, lane 5: 2μL eluate, lane 6: 3μL eluate, lane 7: 1μg BSA, lane 8: 2μL BSA. Molecular mass markers are shown on the left. **(D)** Specificity of ATP filter binding assay: recombinant wild type dMi-2 (+) was incubated with (γ-^{32}P)-ATP. Recombinant dMi-2 was ommitted from some reactions (-) to determine the background binding to the filters. (γ-^{32}P)-ATP (0.93 μM) binding to dMi-2 was competed with increasing concentrations of unlabeled GTP (blue bars; 4.6 μM, 46 μM and 460 μM) or ATP (red bars; 4.6 μM, 46 μM and 460 μM). **(E)** ATP binding by recombinant dMi-2 proteins. The filter binding assay was performed with (γ-^{32}P)-ATP (3000Ci/mmol, 10mCi/mL) and 90nM of dMi-2 proteins as indicated on the bottom. No protein (left) and 90nM IgG (right) were used as negative controls. Radioactivity bound by wild type dMi-2 was set to 100%. Error bars represent s.e.m. and are derived from 3 independent experiments.

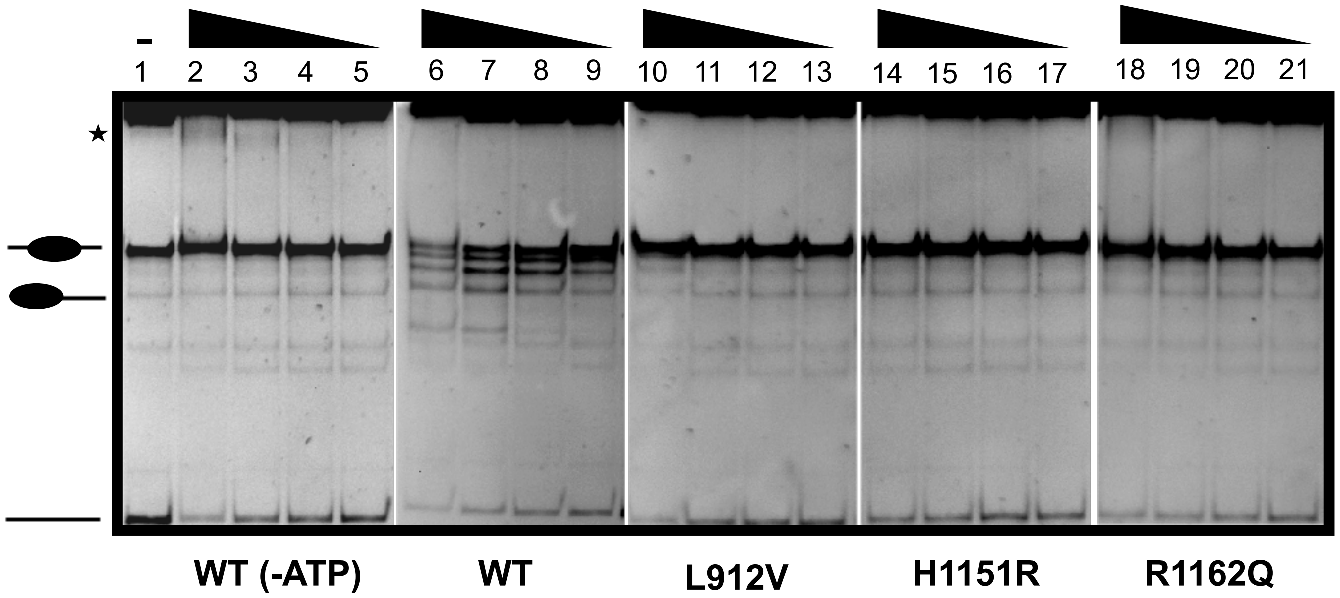

Supplementary Figure 2. Nucleosome sliding assays were carried out with 150 nM of 77-77 mononucleosomes and decreasing concentrations of dMi-2 proteins as indicated (lanes 2, 6, 10, 14, 18: 900 nM; lanes 3, 7, 11, 15, 19: 450 nM; lanes 4, 8, 12, 16, 20: 225 nM; lanes 5, 9, 13, 17, 21: 113 nM). ATP was omitted from reactions shown in lanes 2 to 5 (-ATP). The positions centrally and end positioned mononucleosomes and free DNA are indicated on the left. Asteriks denotes the position of dMi-2/mononucleosome complexes that form at high protein concentrations. All five panels are derived from a single experiment (see Supplementary Figure 10 for the entire gel). Note that we have reproduced the first two panels (WT and WT-ATP) in Supplementary Figures 2, 3 and 5 to aid visual comparison with the activity of mutants analysed in these figures.

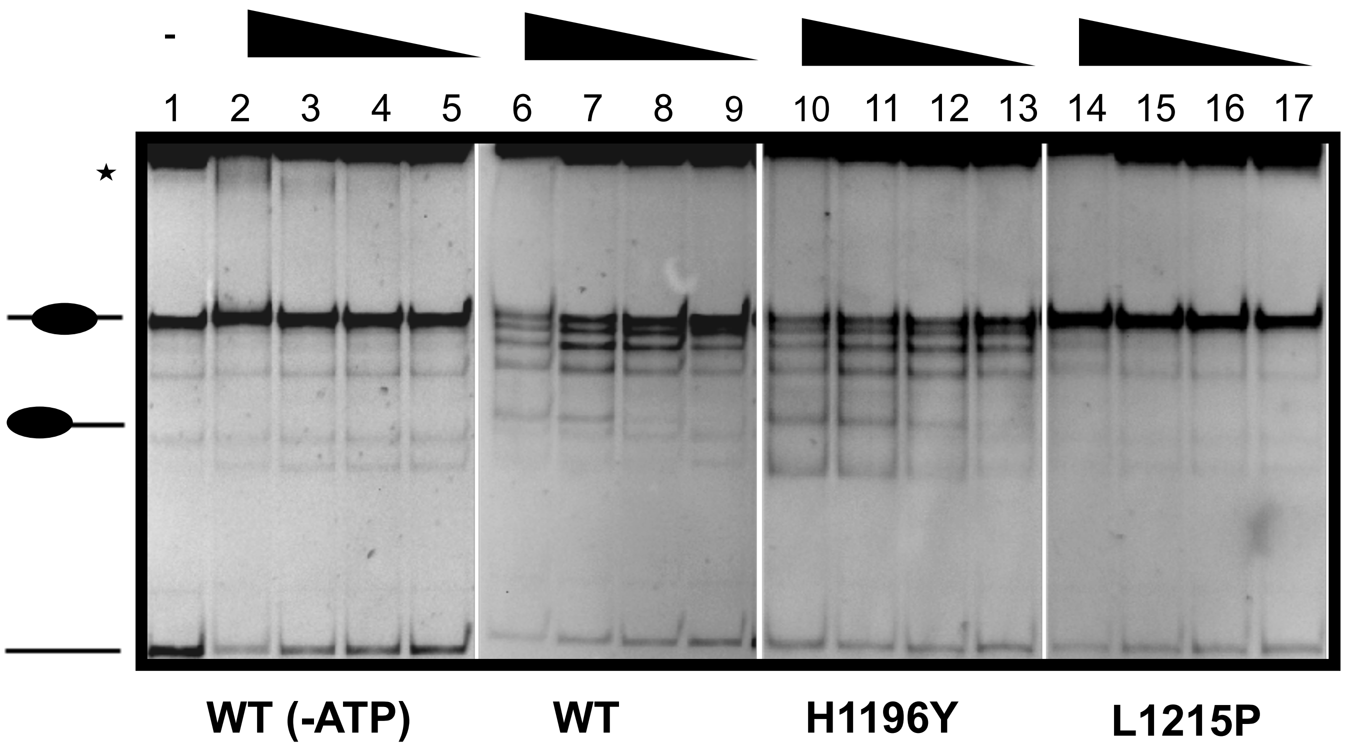

Supplementary Figure 3. Nucleosome sliding assays were carried out with 150 nM of 0-77 mononucleosomes and decreasing concentrations of dMi-2 proteins as indicated. Upper panel: lanes 2, 6, 10, 14: 900 nM; lanes 3, 7, 11, 15: 450 nM; lanes 4, 8, 12, 16: 225 nM; lanes 5, 9, 13, 17: 113 nM). ATP was omitted from reactions shown in lanes 2 to 5(-ATP). The positions centrally and end positioned mononucleosomes and free DNA are indicated on the left. Asteriks denotes the position of dMi-2/mononucleosome complexes that form at high protein concentrations. All panels are derived from same experiment (see Supplementary Figure 10 for the entire gel). Note that we have reproduced the first two panels (WT and WT-ATP) in Supplementary Figures 2, 3 and 5 to aid visual comparison with the activity of mutants analysed in these figures.

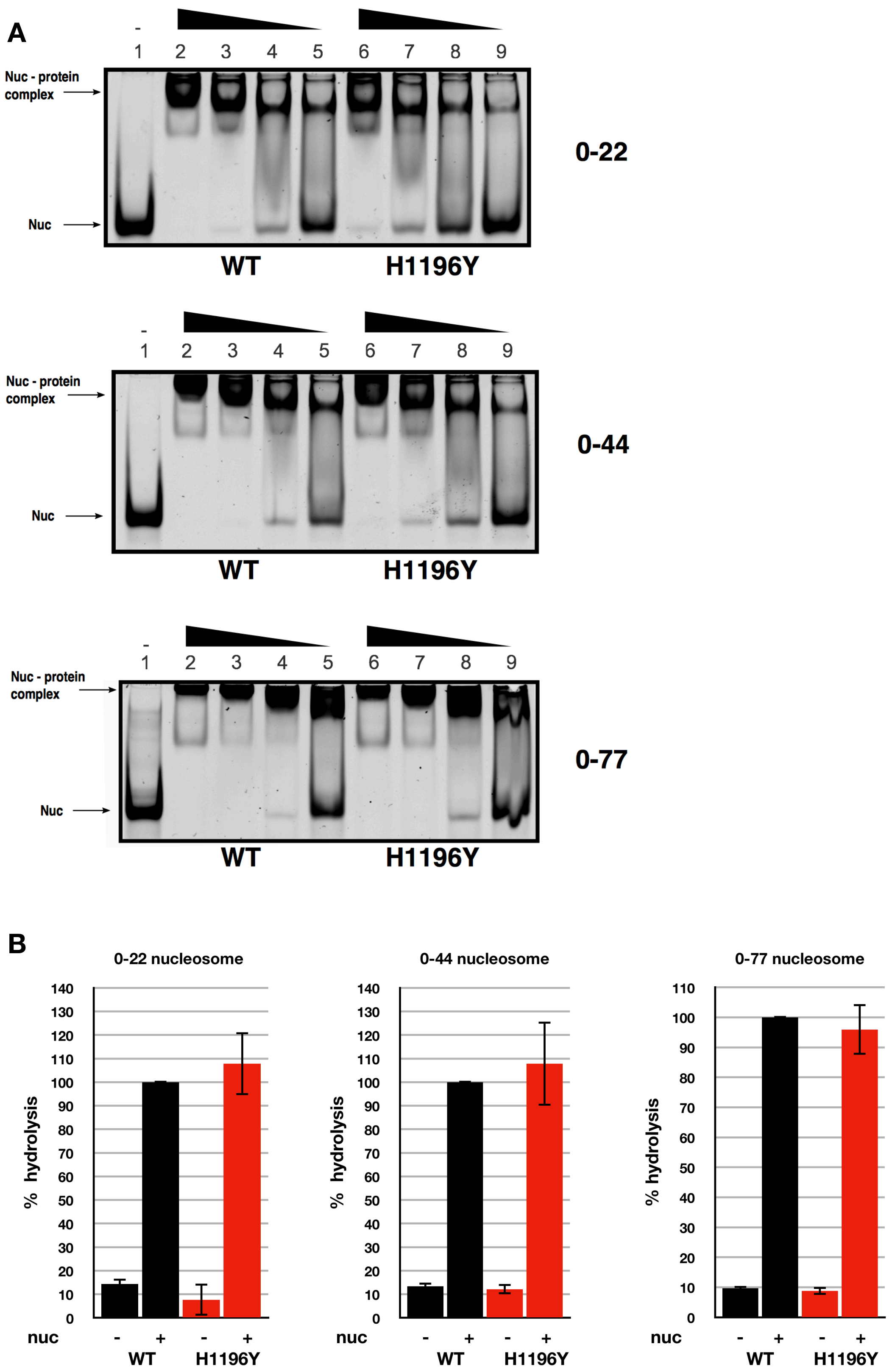

Supplementary Figure 4. (A) Electrophoretic mobility shift assays were carried out with 150 nM of 0-22 mononucleosome (upper panel), 0-44 mononucleosome (middle panel) and 0-77 mononucleosome (lower panel) and decreasing concentrations of dMi-2 proteins as indicated (lanes 2, 6: 900 mM; lanes 3, 7: 450 nM; lanes 4, 8: 225 nM; lanes 5, 9: 113 nM). The positions of nucleosome-protein complexes and unbound mononucleosome are indicated by arrows on the left. **(B)** ATPase activities of dMi-2 WT and dMi-2 H1196Y were determined in absence (-) and presence (+) of saturating amounts of mononucleosomes as indicated (left panel: 0-22 nucleosome, middle panel: 0-44 nucleosome, right panel: 0-77 nucleosome). ATPase activity of wild type dMi-2 was set to 100%. Error bars represent s.e.m. and are derived from 3 independent experiments.

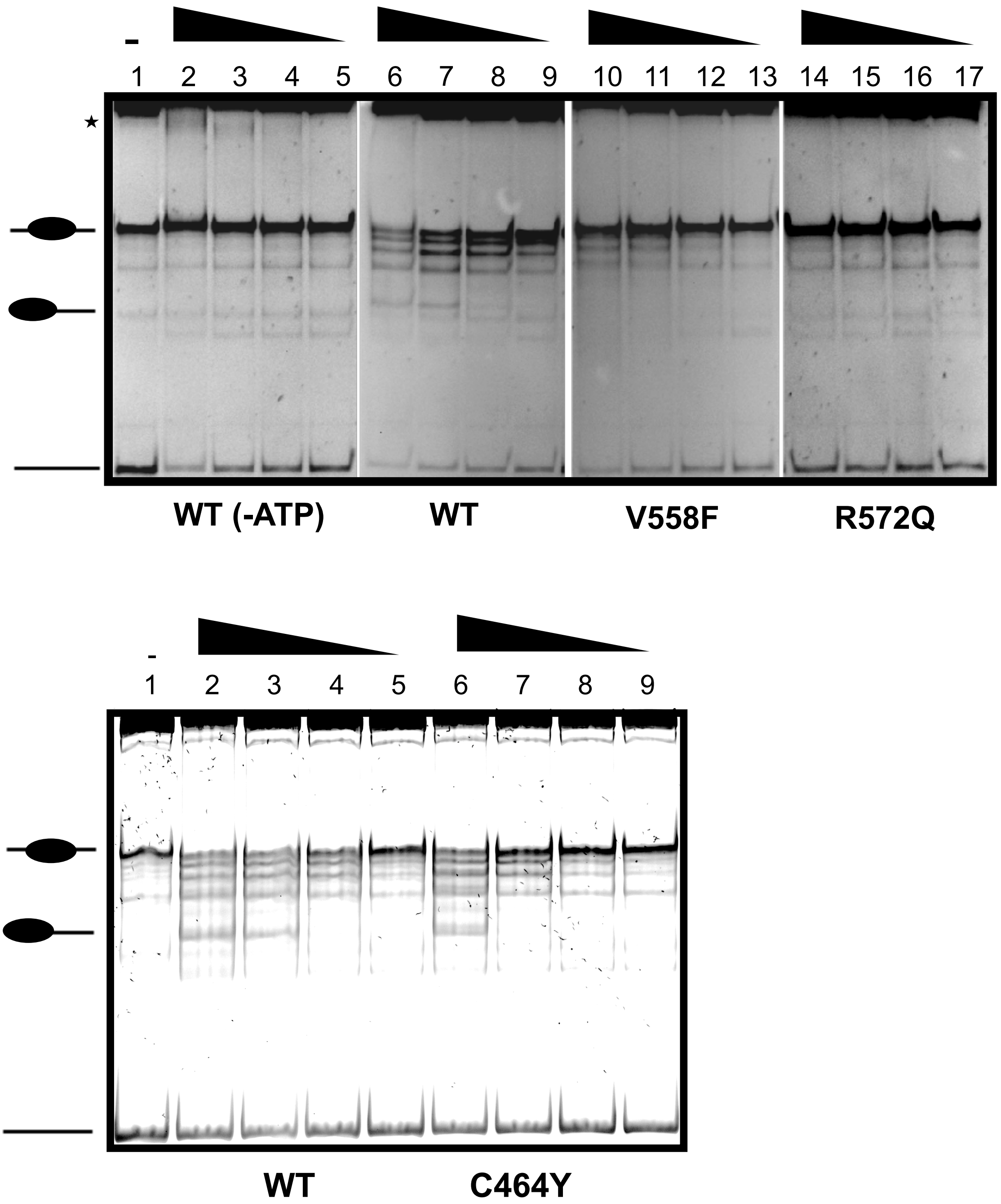

Supplementary Figure 5. Nucleosome sliding assays were carried out with 150 nM of 77-77 mononucleosomes and decreasing concentrations of dMi-2 proteins as indicated (lanes 2, 6, 10, 14: 900 nM; lanes 3, 7, 11, 15: 450 nM; lanes 4, 8, 12, 16: 225 nM; lanes 5, 9, 13, 17: 113 nM). ATP was omitted from reactions shown in lanes 2 to 5 (-ATP). The positions of centrally and end positioned mononucleosomes and free DNA are indicated on the left. Asteriks denotes the position of dMi-2/mononucleosome complexes that form at high protein concentrations. Upper and lower panels show results from two different experiments. The upper panel is part of the larger experiment shown in Supplementary Figure 10. Note that we have reproduced the first two panels (WT and WT-ATP) in Supplementary Figures 2, 3 and 5 to aid visual comparison with the activity of mutants analysed in these figures.

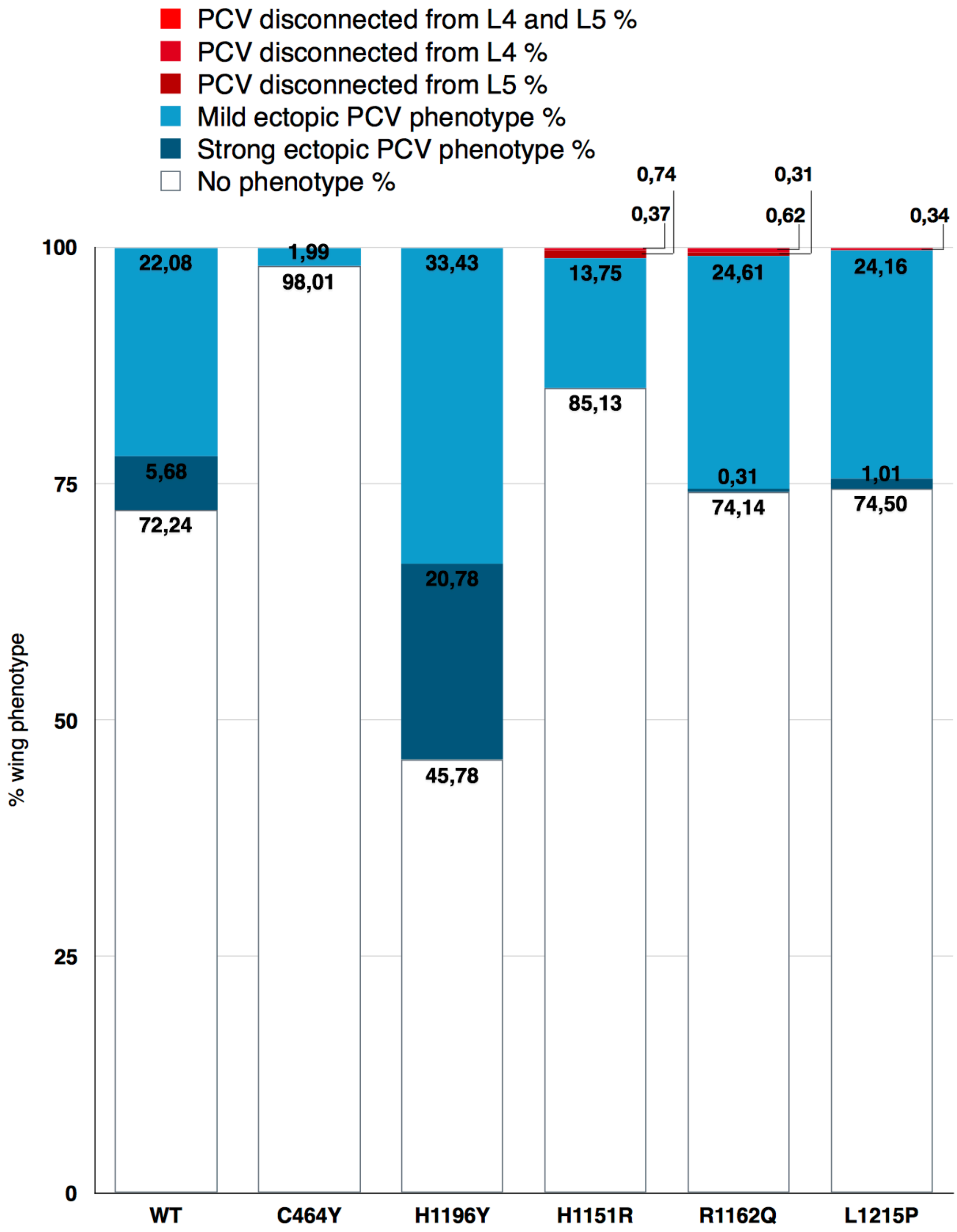

Supplementary Figure 6. Graph showing the distribution (in %) of ectopic PCV (blue) and loss of PCV (red) phenotypes for expression of wild type and mutant dMi-2 proteins at 18^{0}C as indicated.

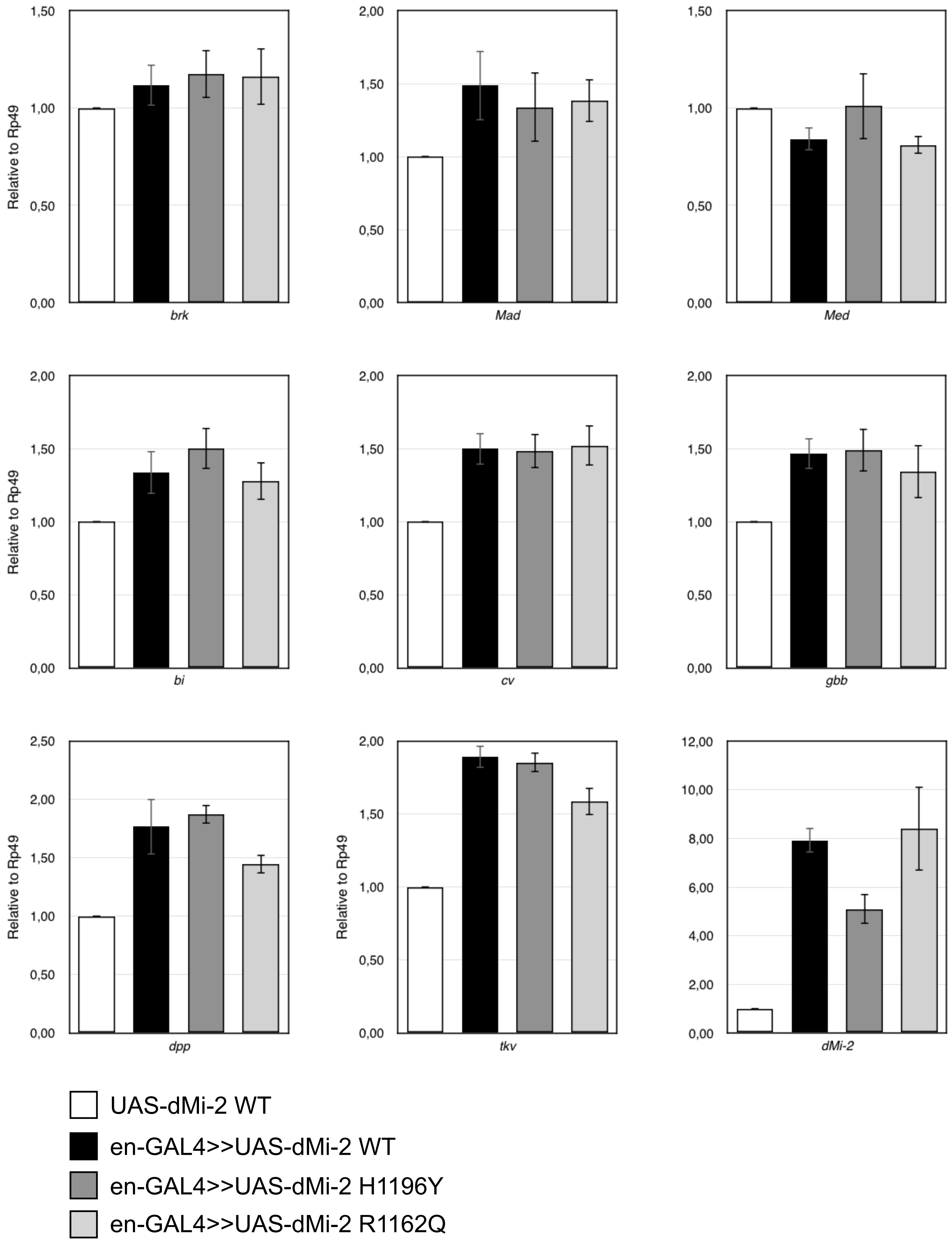

Supplementary Figure 7. RT-qPCR analysis of expression of genes encoding BMP/TGFbeta signaling components. RNA was prepared from whole wing imaginal discs derived from control larvae (*UAS-dMi-2 WT*, a strain carrying the dMi-2 WT transgene but not expressing GAL4) and larvae ectopically expressing dMi-2 WT (*en-GAL4>> UAS-dMi-2 WT*), dMi-2 H1196Y (*en-GAL4>> UAS-dMi-2 H1196Y*) or dMi-2 R1162Q (*en-GAL4>> UAS-dMi-2 R1162Q*). RNA levels in wing discs from control larvae were set to 1. Data shown are mean value and standrad deviation of three different experiments.

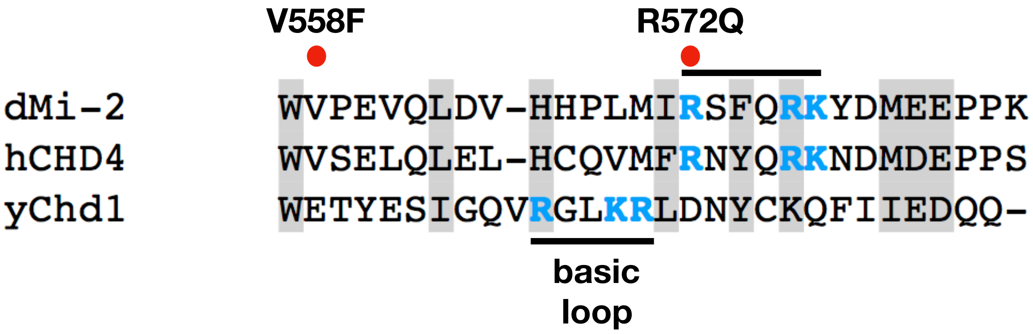

Supplementary Figure 8. Alignment of chromodomain 1 sequences of dMi-2, hCHD4 and yChd1. The basic loop of Chd1 identified by Nodelman and colleagues is underlined. A basic region in dMi-2 and hCHD4 is indicated on top. The positions of the two missense mutations analysed in this study are shown by red dots.

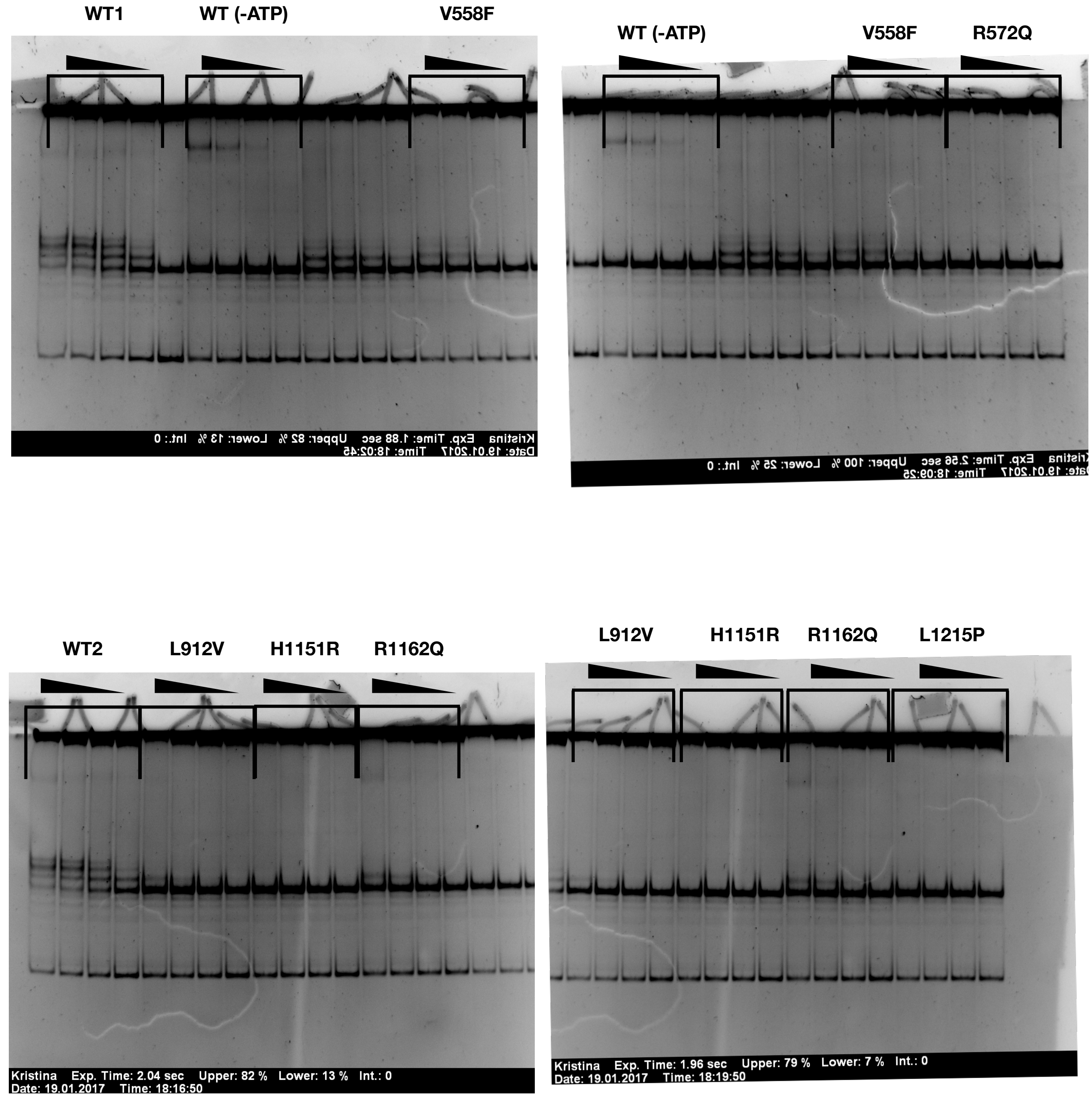

Supplementary Figure 9. Nucleosome sliding assay with 0-77 mononucleosomes. Uncropped gels used to make Figures 2D, 3E and 4D. All reactions are from a single experiment run on two different gels (upper and lower panels). Upper panels and lower panels represent two overlapping photographs of the same gel, respectively. Samples marked with “WT2” were used in Figures 2D, 3E and 4D as “WT” controls.

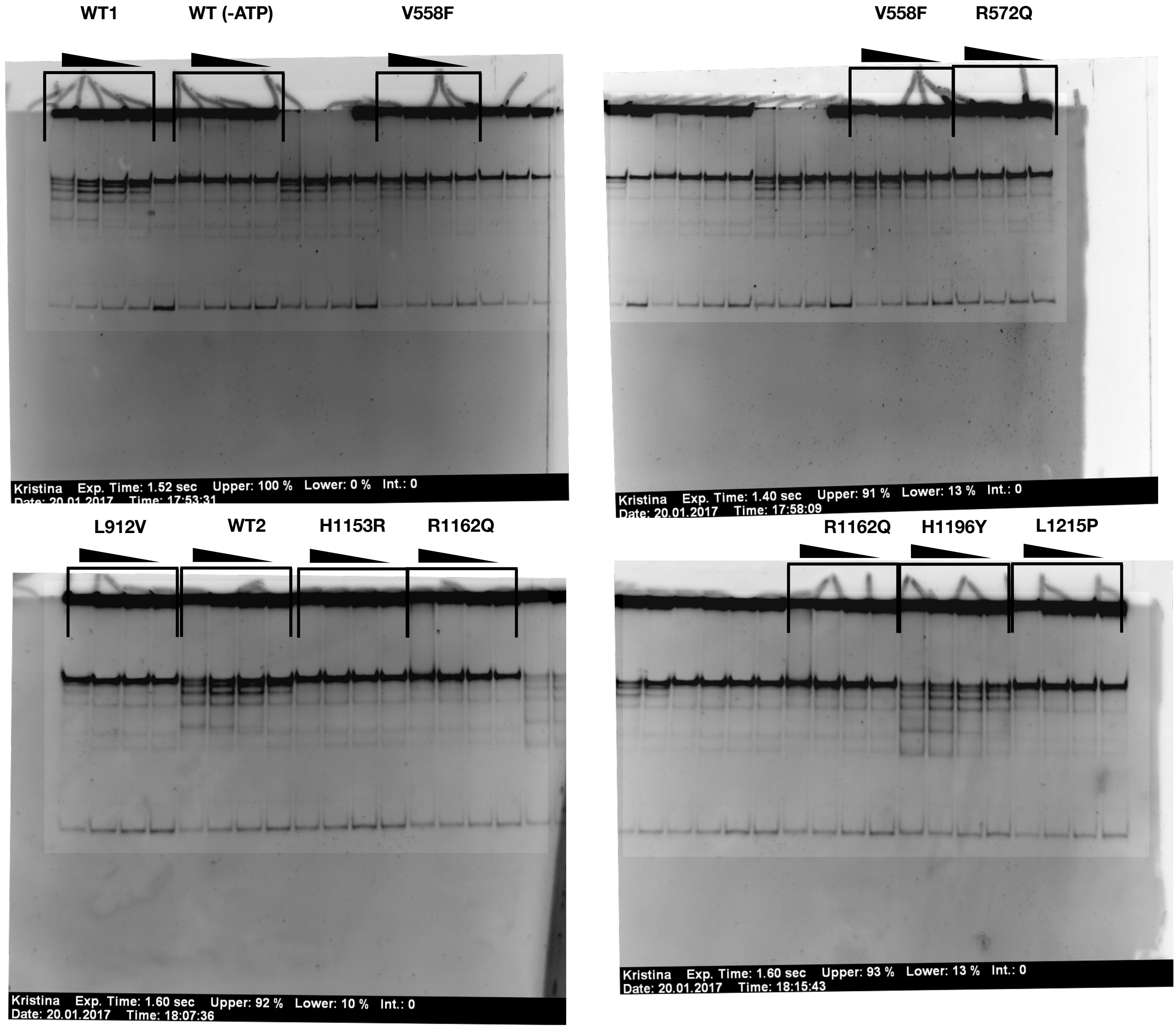

Supplementary Figure 10. Nucleosome sliding assay with 77-77 mononucleosomes. Uncropped gels used to make Supplementary Figures 2, 3 and 5. All reactions are from a single experiment run on two different gels (upper and lower panels). Upper panels and lower panels represent two overlapping photographs of the same gel, respectively. Samples marked with ‘WT2’ were used in Sup Figures 2, 3 and 5 as "WT" controls.
